# Nurse Training in Three Cottage Hospitals: Teignmouth, Dawlish, Newton Abbot

**Published:** 1915-05-29

**Authors:** 


### THE DEPARTMENT OF MODERN NURSING.*

### XIV.—Nurse Training in Three Cottage Hospitals.

### TEIGNMOUTH, DAWLISH, NEWTON ABBOT.

THE first of these three hospitals, that situated at Teignmouth, has accommodation for seventeen patients, but has seldom more than eight or nine beds filled. It is a building adapted from a good-sized country house, which is fortunately surrounded by a beautiful garden and ancient trees. The large halls and corridors make it expensive to keep up, because of the unnecessary spaces to be kept clean; and the patients were, till lately, scattered among the large rooms on two stories in a way that made it impossible for the one nurse on night duty, often a girl of two or three months' experience, to have much supervision over them.

The staff consists of a matron, nurse, and two probationers. The latter are received for a year, though, in case of their being suitable and wishing to stay longer, no objection is made. No certificate is given, and most of those trained here come only for preliminary instruction to fill a gap until they are old enough for a larger hospital. The age for entering is nineteen or twenty. The two probationers take day and night duty alternately, doing it for two months at a time. The salary for the year is £8. There are about two operations a week, at which the probationer on day duty is present to help. There are also a few out-patients, principally eye cases, which the probationer has to attend. As cases received are mostly chronic, there is little material for teaching probationers; and there is a good deal of house-work to be done, although two servants are kept and a charwoman employed frequently.

The second hospital visited, that at Dawlish, was a much smaller building, but it had been erected for a hospital instead of being adapted from an old house. It contains two wards with four beds and a cot in each, and two private rooms, each with one bed. The wards have balconies, of which much use is made. The average number of patients is from six to eight, though it happens sometimes that for weeks together there may be only one bed occupied. But it is a busy little hospital, and these slack times are well filled up in doing stock work, sewing, etc., for which, when the beds are full, there is often no time at all.

The staff is composed of a nurse-matron and two probationers. The latter are nominally engaged for a year, but if possible they are kept on a few months longer, and then, as a rule, enter for three years at a general hospital. No certificate is given. The hours are from 7 A.M. to 10 P.M., with two hours off daily and an hour allowed for each mealtime. A very occasional whole day is given, and a fortnight's holiday in the year, which the matron always lengthens if possible. No salary is given during the first year, but uniform is found; after the first year the rate of payment is £10 without uniform. There is no night duty, but the senior probationer gets up in the night if it is required. For a patient needing night attendance a special nurse is obtained.

A good deal of surgery is done here, four operations in a week being quite usual. Of course, many cases are trivial, but there is a fair amount of abdominal surgery done, and the probationers as a rule do the dressings. They also look after the out-patients, of whom there are a few daily. The two probationers have each a comfortable little room to themselves. There is no teaching for them, except the practical daily work.

The third cottage hospital, situated at Newton Abbot, is on a much larger scale, an average of twenty-five out of the forty beds being in daily use. The matron has under her one sister, trained elsewhere, three private nurses, who work in the wards when they are not employed at private cases, and four probationers. On night duty there is one senior probationer and a junior. No nurse, however, goes on night duty as senior till she has had six months' day duty. One month in three is generally spent on night work.

Though there is an article in the agreement that probationers may be required to take private cases outside the hospital for the third year, at present nurses have the whole three years in the wards.

The hours of duty are from 7.20 A.M. to either 8 or 9.30 P.M. There is a half-hour's interval in the middle of the morning and the same for dinner and tea. Each nurse is also off duty daily for either two and a-half hours in the morning or two hours in the afternoon, with an extra hour on Sunday, and a whole day every month. Three weeks' holiday in the year is given, and it is preferred that it should be taken in two parts.

The nominal age of candidates is from twenty-one to thirty, but probationers of nineteen and twenty are more usual. The salaries are £8, £10, £20, and £25 for the four years. Indoor uniform only is provided during training. Lectures are given by the honorary staff on anatomy, physiology, medicine and surgery. Classes are also given; and the examinations held, first by the teacher, and at the end of the three years by an outside doctor, must be passed before a certificate is obtained.

There is no massage teaching at present, no dispensing, and no cookery lessons. There is some training in *x*-ray work, and some gynæcological work. Each nurse has a turn in the theatre, and in the absence of any resident medical staff there is good experience to be gained.

The agreement signed by the candidate provides that she shall not work as private nurse within the urban district for five years after leaving the hospital under a penalty of £25. A formal three years' certificate of training is given.

* Previous articles: May 16, 30; June 13; July 11, 25; Aug. 8, 1914; Jan. 16, 30; Feb. 27; March 13; April 3; May 8 and 22, 1915.

